# Supplementary material for: A Novel Strategy to Reveal the Landscape of Crossovers in an F1 Hybrid Population of Populus deltoides and Populus simonii
Source: Plants (Basel). 2022 Apr 12;11(8):1046. doi: 10.3390/plants11081046 (PMC9025136; doi:10.3390/plants11081046)
Supplement: Supplementary file 1 [file plants-11-01046-s001.zip › SuppFileS2.pdf]

Text S1. Calculation of the coefficient of coincidence between the two adjacent SNP intervals for the female parent in an F_1 population of *Populus deltoides* and *Populus simonii*

Considering three ordered SNPs of 1, 2, and 3 that segregate in *ab*×*aa*, the counts and frequencies of the genotypes at the three SNPs in an F_1 hybrid population of *P. deltoides* and *P. simonii* under different linkage phases are listed in Table S2, where r_{ij} denotes the frequency of crossovers that occur (i = 1) or not (i = 0) in the first interval between SNPs 1 and 2 and that occur (j = 1) or not (j = 0) in the second interval between SNPs 2 and 3.

Let C denote the coefficient of coincidence between the first and second intervals. Then, under the linkage phase of case 1 (Table S2), the crossover frequencies can be expressed as following

$$\begin{cases} r_{11} = r_1 r_2 C \\ r_{01} = r_2 - r_1 r_2 C \\ r_{10} = r_1 - r_1 r_2 C \\ r_{00} = 1 - r_1 - r_2 + r_1 r_2 C \end{cases} \quad \text{(S1)}$$

where r_1 and r_2 are the recombination fractions of the first and second intervals, respectively. When r_1 and r_2 are known, the likelihood of the coefficient of coincidence can be written as

$$L(C) = \frac{n!}{\prod_{j=1}^{8} n_j!} \left(\frac{1}{2} r_{00}\right)^{n_1+n_8} \left(\frac{1}{2} r_{01}\right)^{n_2+n_7} \left(\frac{1}{2} r_{10}\right)^{n_4+n_5} \left(\frac{1}{2} r_{11}\right)^{n_3+n_6} \quad \text{(S2)}$$

where $n = n_1 + n_2 + \cdots + n_8$.

The derivatives of the log-likelihood with respective to r_1, r_2, and C can be derived to be

$$\begin{cases} \dfrac{\partial \ln L}{\partial r_1} = \dfrac{n_3+n_6}{r_1} + \dfrac{n_4+n_5}{r_1} - \dfrac{(n_2+n_7)C}{1-r_1C} - \dfrac{(n_1+n_8)(1-r_2C)}{1-r_1-r_2+r_1r_2C} \\ \dfrac{\partial \ln L}{\partial r_2} = \dfrac{n_3+n_6}{r_2} - \dfrac{(n_4+n_5)C}{1-r_2C} + \dfrac{n_2+n_7}{r_2} - \dfrac{(n_1+n_8)(1-r_1C)}{1-r_1-r_2+r_1r_2C} \\ \dfrac{\partial \ln L}{\partial C} = \dfrac{n_3+n_6}{C} - \dfrac{(n_4+n_5)r_2}{1-r_2C} - \dfrac{(n_2+n_7)r_1}{1-r_1C} + \dfrac{(n_1+n_8)r_1r_2}{1-r_1-r_2+r_1r_2C} \end{cases} \quad \text{(S3)}$$

If we set the derivatives above to be zero, then the MLEs of r_1, r_2, and C can be solved as following

$$\begin{cases} \hat{r}_1 = \dfrac{1}{n}\left(n_3+n_4+n_5+n_6\right) \\ \hat{r}_2 = \dfrac{1}{n}\left(n_2+n_3+n_6+n_7\right) \\ \hat{C} = \dfrac{n_3+n_6}{n\hat{r}_1\hat{r}_2} \end{cases} \quad \text{(S4)}$$

For the other cases of linkage phase (Table S2), the MLE of the parameters can be similarly derived as following,

$$\begin{cases} \hat{r}_1 = \dfrac{1}{n}\left(n_3+n_4+n_5+n_6\right) \\ \hat{r}_2 = \dfrac{1}{n}\left(n_1+n_4+n_5+n_8\right) \\ \hat{C} = \dfrac{n_4+n_5}{n\hat{r}_1\hat{r}_2} \end{cases} \quad \text{for case 2} \quad \text{(S5)}$$

$$\begin{cases} \hat{r}_1 = \frac{1}{n}\left(n_1 + n_2 + n_7 + n_8\right) \\ \hat{r}_2 = \frac{1}{n}\left(n_1 + n_4 + n_5 + n_8\right) \\ \hat{C} = \frac{n_1 + n_8}{n\hat{r}_1\hat{r}_2} \end{cases} \quad \text{for case 3} \qquad \text{(S6)}$$

$$\begin{cases} \hat{r}_1 = \frac{1}{n}\left(n_1 + n_2 + n_7 + n_8\right) \\ \hat{r}_2 = \frac{1}{n}\left(n_2 + n_3 + n_6 + n_7\right) \\ \hat{C} = \frac{n_2 + n_7}{n\hat{r}_1\hat{r}_2} \end{cases} \quad \text{for case 4} \qquad \text{(S7)}$$

Table S2. The counts and frequencies of genotypes at SNPs 1, 2, and 3 that segregate in *ab*×*aa* under different linkage phases.

Linkage Phase				Genotype SNPs 1 2 3	Count	Frequency
Case 1				*aa aa aa*	n_1	$r_{00}/2$
				aa aa ab	n_2	$r_{01}/2$
SNP 1	*a* | *b*		*b* | *a*	*aa ab aa*	n_3	$r_{11}/2$
SNP 2	*a* | *b*	or	*b* | *a*	*aa ab ab*	n_4	$r_{10}/2$
SNP 3	*a* | *b*		*b* | *a*	*ab aa aa*	n_5	$r_{10}/2$
				ab aa ab	n_6	$r_{11}/2$
				ab ab aa	n_7	$r_{01}/2$
				ab ab ab	n_8	$r_{00}/2$
Case 2				*aa aa aa*	n_1	$r_{01}/2$
				aa aa ab	n_2	$r_{00}/2$
SNP 1	*a* | *b*		*b* | *a*	*aa ab aa*	n_3	$r_{10}/2$
SNP 2	*a* | *b*	or	*b* | *a*	*aa ab ab*	n_4	$r_{11}/2$
SNP 3	*b* | *a*		*a* | *b*	*ab aa aa*	n_5	$r_{11}/2$
				ab aa ab	n_6	$r_{10}/2$
				ab ab aa	n_7	$r_{00}/2$
				ab ab ab	n_8	$r_{01}/2$
Case 3				*aa aa aa*	n_1	$r_{11}/2$
				aa aa ab	n_2	$r_{10}/2$
SNP 1	*a* | *b*		*b* | *a*	*aa ab aa*	n_3	$r_{00}/2$
SNP 2	*b* | *a*	or	*a* | *b*	*aa ab ab*	n_4	$r_{01}/2$
SNP 3	*a* | *b*		*b* | *a*	*ab aa aa*	n_5	$r_{01}/2$
				ab aa ab	n_6	$r_{00}/2$
				ab ab aa	n_7	$r_{10}/2$
				ab ab ab	n_8	$r_{11}/2$
Case 4				*aa aa aa*	n_1	$r_{10}/2$
				aa aa ab	n_2	$r_{11}/2$
SNP 1	*a* | *b*		*b* | *a*	*aa ab aa*	n_3	$r_{01}/2$
SNP 2	*b* | *a*	or	*a* | *b*	*aa ab ab*	n_4	$r_{00}/2$
SNP 3	*b* | *a*		*a* | *b*	*ab aa aa*	n_5	$r_{00}/2$
				ab aa ab	n_6	$r_{01}/2$
				ab ab aa	n_7	$r_{11}/2$
				ab ab ab	n_8	$r_{10}/2$
